# Telemedicine screening for syphilitic chorioretinitis in the SUNDROP cohort

**DOI:** 10.1038/s41433-022-01967-x

**Published:** 2022-02-25

**Authors:** Moosa H. Zaidi, Tatiana R. Rosenblatt, Ahmad Al-Moujahed, Daniel Vail, Natalia F. Callaway, Quan Dong Nguyen, Darius M. Moshfeghi

**Affiliations:** Byers Eye Institute, Stanford University School of Medicine, Palo Alto, CA USA

**Keywords:** Infectious diseases, Retinal diseases, Uveal diseases, Epidemiology, Risk factors

## Introduction

Chorioretinitis is a serious ocular complication of congenital syphilis (CS) that can cause permanent vision loss. While antibiotic use and public awareness have helped dramatically reduce the incidence of syphilis in the twentieth century, the US incidence of syphilis has more than tripled from 2000 to 2018 [1, 2]. Although the preponderance of syphilitic infections during this period have been in males, the incidence of primary and secondary syphilis in females has also risen from 1.7 per 100,000 in 2000 to 3.0 per 100,000 in 2018. In parallel, the incidence of CS has risen from 14.1 per 100,000 births in 2000 to 32.8 per 100,000 births in 2018 [2]. These trends raise a concern about the incidence of congenital syphilitic chorioretinitis. Yet, to the authors’ knowledge, no recent studies have examined this incidence.

## Methods

The Stanford University Network for Diagnosis of Retinopathy of Prematurity (SUNDROP) study is a telemedicine screening platform for retinopathy of prematurity (ROP) used by member hospitals that lack access to quaternary pediatric retina specialists. The SUNDROP platform has been used in expanded indications beyond ROP to provide hospitals with pediatric retinal screening including the transmission of retinal images in infants with known or suspected CS. We retrospectively analyzed the SUNDROP database to examine a more recent incidence of chorioretinitis among cases of CS. This index study was conducted in accordance with the Health Insurance Portability and Accountability Act and the tenets of the Declaration of Helsinki and with local approval of the Institutional Board Review (IRB 8572).

## Results

Thirty-seven confirmed and seven probable cases of CS were identified from 2015 to 2020 following Centers for Disease Control case definitions [3]. An additional 17 cases of rapid plasma reagin (RPR) positivity alone were also identified. Across all 61 of these cases, no evidence of chorioretinitis was found based on fundus photographic findings. The overall sex distribution was 44.3% female, including 40.5% of confirmed CS cases, 28.6% of probable CS cases, and 90.9% of positive RPR titer-only cases.

## Discussion

These preliminary findings suggest that even as the incidences of CS have risen, chorioretinitis remains a rare complication. While these results are specific to CS in neonates, they are consistent with a recent study that found hospital admissions for syphilitic uveitis have not increased despite a growing incidence of admissions for syphilis [4]. Although every case of CS results from inadequate maternal treatment, it may be that late or partial maternal treatment results in milder cases of CS with a lower incidence of chorioretinitis than cases resulting from no maternal treatment. Indeed, the most recent prior study of the incidence of chorioretinitis in CS conducted in 1953 noted that chorioretinitis, as well as other symptoms signs such as dacryocystitis and Hutchinson triad, appeared to have become rarer and less severe in CS [5]. Thus, the low incidence of chorioretinitis presently observed may reflect both its rarity in the natural history of CS and the effect of partial maternal treatment.
